# Potentially inappropriate medications among older patients with Parkinson’s disease: a cross-sectional analysis of a national health insurance database in China

**DOI:** 10.1186/s12877-023-04547-0

**Published:** 2023-12-08

**Authors:** Mengyuan Fu, Can Li, Zinan Zhao, Kexin Ling, Zhiwen Gong, Huangqianyu Li, Ting Li, Jianchun Li, Weihang Cao, Xin Hu, Luwen Shi, Pengfei Jin, Xiaodong Guan

**Affiliations:** 1 Department of Pharmacy Administration and Clinical Pharmacy, School of Pharmaceutical Sciences, Peking University, Beijing, People’s Republic of China; 2 International Research Center for Medicinal Administration, Peking University, Beijing, People’s Republic of China; 3 Department of Pharmacy, Beijing Hospital), Beijing, People’s Republic of China; 4 National Center of Gerontology, Beijing, People’s Republic of China; 5 Institute of Geriatric Medicine, Chinese Academy of Medical Sciences, Beijing, People’s Republic of China; 6 Beijing Key Laboratory of Assessment of Clinical Drugs Risk and Individual Application, Beijing Hospital), Beijing, 100730 People’s Republic of China

**Keywords:** Parkinson’s disease, Potentially inappropriate medication, PIM, Motor impairment, Cognitive impairment

## Abstract

**Background:**

With the rapid aging trend of China's population, the issue of drug rational use in older adults has become more and more prominent. Parkinson’s disease (PD) is the one of the most common age-related neurodegenerative disorders. Pharmaceutical treatment plays a cardinal role in alleviating motor and non-motor symptoms to improve the quality of life of patients with PD. Patients with PD have complex medical needs yet little is known about the use of potentially inappropriate medications (PIM) among them in China. We quantify the prevalence of PIM use and identify its predictors among older persons with PD in China.

**Methods:**

We conducted a cross-sectional analysis using a national representative database of all medical insurance beneficiaries across China, extracting records of ambulatory visits of older adults with PD between 2015 and 2017. Beneficiaries aged 65 and above were eligible for inclusion. The prevalence of patients exposed to overall PIMs and PIMs related to motor and cognitive impairment was calculated based on Beers Criteria 2015 version. Potential predictors of PIM concerning patients’ characteristics were estimated using multivariate logistic regression.

**Results:**

A total of 14,452 older adults with PD were included. In total, 8,356 (57.8%) patients received at least one PIM; 2,464 (17.1%) patients received at least one motor-impairing PIM and 6,201 (42.9%) patients received at least one cognition-impairing PIM. The prevalence of overall PIM use was higher in patients of older age group (54.7% [65–74] *vs.* 59.5% [75–84; OR, 1.22; 95% CI, 1.14–1.31] *vs*.65.5% [≥ 85; OR, 1.58; 95% CI, 1.38–1.80) and females (61.4% [female] *vs.* 55.0% [males; OR, 0.77; 95% CI, 0.72–0.82).

**Conclusions:**

Prescribing PIMs for older adults with PD was common in China, especially for females and older age groups, yet younger patients were more inclined to be prescribed with motor or cognition-impaired PIMs. Our findings represent a clear target awaiting multidimensional efforts to promote the rational prescribing of medications for this vulnerable population.

**Supplementary Information:**

The online version contains supplementary material available at 10.1186/s12877-023-04547-0.

## Introduction

Parkinson’s disease (PD) is the one of the most common age-related neurodegenerative disorders [1, 2], the incidence of which sharply increases after 60 years of age [3]. While the progress of motor impairment can be taken under control with antiparkinsonian drugs, cognitive impairment, such as cognitive abnormalities, autonomic dysfunction, sleep disorders, and mood disorders, affecting 75% of PD patients who had a PD disease course of more than ten years, is deemed as one of the most distressing complications of PD for patients and their caregivers [4–8]. Alleviating cognitive symptoms using pharmaceutical treatment plays an extremely significant role to promote the quality of life of PD patients [7, 8]. However, prescribing medications for patients with PD needs extra deliberations as adverse drug-drug and drug-disease interactions can exacerbate PD symptoms and aggravate healthcare burden [9]. Hence, it is of great importance to evaluate the appropriateness of medication use among PD patients. The American Geriatrics Society (AGS) Beers criteria list potentially inappropriate medications (PIMs) that should be avoided or used with caution among older patients [10–12]. Previous studies observed that 35.8% of older patients with PD were exposed to PIMs in the US [13] and patients using PIMs had longer duration of hospital stay [14].

China has a rapidly ageing population [15] and 1.7% of older adults aged 65 years and above were diagnosed with PD [16]. It is estimated that the number of PD patients in China will reach 4.94 million by 2030, accounting for half of the global figure [17]. Though studies have evaluated PIM prescribing for the general older population in China [16, 17], older adults living with PD experience even heightened health risks due to their complex medical needs and higher susceptibility to PIM use. However, there is scarce literature investigating PIM use in this population in China and studies from abroad, mainly conducted in the United States, were unlikely to be generalizable to the Chinese context. Our study aimed to quantify the prevalence of PIM use and identify its risk factors among the older individuals with PD in China.

## Methods

### Study design

We conducted a cross-sectional study to explore the overall prevalence of PIM use and the prevalence of PIMs that might cause motor or cognitive impairments among older persons with PD across China, using a national representative dataset of claims data from 2015 to 2017.

### Financial coverage of Chinese health insurance scheme

By the end of 2015, over 95% of the Chinese population were covered by one of the two basic medical insurance (BMI) schemes [18]. The Urban Rural Resident Basic Medical Insurance (URRBMI) covers the urban non-employed and self-employed population and rural residents while the Urban Employee Basic Medical Insurance (UEBMI) covers the urban employees and the retired people [19]. The financial coverage of URRBMI and UEBMI are different. The UEBMI entails a lower deductible and higher reimbursement ratio and cap [20].

### Data source and data collection

The China Health Insurance Association (CHIRA) collects all medical records of a national representative sample (sample ratio 2%) of all BMI beneficiaries across China every year, using a systematic random sampling strategy elaborated in previous studies [21–25]. We extracted all records of ambulatory visit of older beneficiaries (aged 65 years and above) between 2015 and 2017 from the CHIRA database, including information about the patient’s age, gender, type of insurance scheme, province of residence, visit date, diagnoses, and medications prescribed. All data were digitally transferred and verified.

### Inclusion criteria

The CHIRA database coded diagnoses based on the World Health Organization (WHO) International Classification of Diseases Tenth Revision (ICD-10) [26] and drugs according to the WHO Anatomical Therapeutic Chemical (ATC) classification system [27]. We sorted PD diagnosis according to the ICD-10. Patients who had at least one record of ambulatory visit, a diagnosis of PD (ICD-10 code G20), and no record of atypical parkinsonism diagnosis (ICD-10 code G21) during the study period were eligible for inclusion regardless of whether they were prescribed with anti-Parkinson drugs (N04) or not during observation periods.

### Outcome measures

We used the 2015 version of the AGS Beers criteria to evaluate the incidence of PIMs [10]. In this study, PIMs were defined as medications that: 1) are best avoided in most circumstances; 2) should be used with caution; 3) are best avoided in PD patients due to drug-PD interactions; and 4) are involved in drug-drug interactions listed in the Beers criteria. Some entries of the Beers criteria that were not measured are shown in Table S1. Medications with a strong tendency to worsen motor symptoms of PD, including antiparkinsonian agents to be avoided in most conditions and agents that should be avoided due to drug-PD interaction listed in the Beers criteria, were defined as motor-impairing PIMs (Table S2). Medications that would potentially exacerbate cognitive impairment experienced by PD patients, including agents to be avoided in most conditions and agents that should be avoided due to drug-dementia interaction listed in the Beers criteria, were defined as cognition-impairing PIMs (Table S3). Aripiprazole was deemed as a PIM due to the classification change from the 2015 to 2023 Beers criteria [11].

The primary outcome was the proportion of PD patients that were exposed to at least one PIM. Secondary outcomes were the proportions of PD patients that were exposed to motor-impairing PIMs or cognition-impairing PIMs. We also used the 2023 updated version of Beers Criteria in the sensitive analysis [11].

### Statistical analysis

We conducted descriptive analyses to show patients’ characteristics and PIM use. We applied the generalized linear model with logit link function and binomial distribution to examine the adjusted associations between the use of PIMs and independent variables, including year and patients’ age, gender, type of insurance scheme, and region of residence. We classified 31 provinces in mainland China into three regions based on geographic distribution, economic, and other environmental factors (Table S4). The marginal standardization approach adjusted by generalized linear model with logit link function and binomial distribution was applied to calculate the mean adjusted rates of PIM, where they were summed to a weighted average that reflected the population distribution across regions in China. We conducted statistical analyses using Stata version 17. The level of statistical significance was defined as *P* < 0.05 (two-sided).

## Results

### Study sample and characteristics

Of the 2,545,430 older adults aged 65 years and above identified in the CHIRA database between 2015 and 2017, 14,452 (0.6%) were with a PD diagnosis, who filed 118,478 ambulatory prescriptions and had a median (interquartile range [IQR]) age of 76 (70–81) years. PD patients were mostly below 85 years of age, with 6,603 (45.7%) patients aging 65 to 74 years and 6,705 (46.4%) patients aging 75 to 84 years. Most of PD patients were male (8,037 [55.6%]), with UEBMI (12,215 [84.5%]), and residing in the eastern region of China (5,836 [43.5%], Table 1).

**Table 1:** Characteristics and overall PIM use of older adults with Parkinson’s disease in China, 2015–2017 (*n* = 14,452)

Characteristic	Sample size (n, %)	PIM exposure	PIM rate (%)	Adjusted rate ^a^	OR	95% CI	
	14,452 (100)	8,356	57.8	57.8	-	-	-
**Age**							
65–74	6,603 (45.7)	3,650	55.3	54.7	ref	ref	ref
75–84	6,705 (46.4)	3,958	59.0	59.5	1.22^***^	1.14	1.31
≥ 85	1,144 (7.9)	748	65.4	65.5	1.58^***^	1.38	1.80
**Gender**							
Female	6,415 (44.4)	3,975	62.0	61.4	ref	ref	ref
Male	8,037 (55.6)	4,381	54.5	55.0	0.77^***^	0.72	0.82
**Insurance type**							
URRBMI	2,237 (15.5)	1,522	68.0	67.3	ref	ref	ref
UEBMI	12,215 (84.5)	6,834	55.9	56.1	0.62^***^	0.56	0.68
**Region**							
East	6,292 (43.5)	3,788	60.2	59.9	ref	ref	ref
Central	4,596 (31.8)	2,696	58.7	58.5	0.94	0.87	1.02
West	3,564 (24.7)	1,872	52.5	53.2	0.76^***^	0.70	0.82

*Abbreviations*: *PIM* potentially inappropriate medication, *OR* Odds ratio, *95% CI* 95% confidence interval, *URRBMI* Urban Rural Resident Basic Medical Insurance, *UEBMI* Urban Employee Basic Medical Insurance

^a^adjusted by generalized linear model with logit link function and binomial distribution, using marginal standardization approach

^*^p < 0.05

^**^p < 0.01

^***^p < 0.001

### Overall PIM prescribing for older adults with PD

Among all older adults with PD, 8,356 (57.8%) had at least one prescription containing PIM during our study period. After adjustment, the prevalence of PIM use increased with age and was significantly higher in patients who were ≥ 85 years of age (65.5%; OR, 1.58; 95% CI, 1.38–1.80) and lower in males (55.0%; OR, 0.77; 95% CI, 0.72–0.82). The odds of receiving a prescription containing a PIM was significantly lower for patients with UEBMI (56.1%; OR, 0.62; 95% CI, 0.56–0.68) and residing in the western region (53.2%; OR, 0.76; 95% CI, 0.70–0.82, Table 1). The overall PIM rate was 63.4% based on the 2023 Beers Criteria, which is slightly higher than that observed using the 2015 version; yet the influencing factors were relatively stable. ( Table S5).

### Motor-impairing PIMs for older adults with PD

Among all the older persons with PD, 2,464 (17.1%) patients received at least one PIM that could worsen PD motor symptoms. Patients’ age, gender, insurance type, and region of residence were significantly associated with the possibility of receiving motor-impairing PIMs. Contrary to the overall prevalence of PIM use, patients who aged 65–74 years were at higher odds of receiving motor-impairing PIMs (19.4%, reference group), compared with patients who aged 75–84 (14.6%, OR, 0.71; 95% CI, 0.64–0.78) or > 84 (17.7%; OR, 0.90; 95% CI, 0.76–1.06) years (Table 2). The associations other than age group were in the same direction as for overall PIM use.

**Table 2:** Factors associated with receipt of any motor-impairing PIMs among older adults with Parkinson’s disease in China, 2015–2017 (*n* = 14,452)

Characteristic	PIM exposure	PIM rate (%)	Adjusted rate ^a^	OR	95% CI	
**Total**	2,464	17.0	17.1	-	-	-
**Age**						
65–74	1,302	19.7	19.4	ref	ref	ref
75–84	962	14.3	14.6	0.71^***^	0.64	0.78
≥ 85	200	17.5	17.7	0.90	0.76	1.06
**Gender**						
Female	1,315	20.5	20.0	ref	ref	ref
Male	1,149	14.3	14.6	0.68^***^	0.62	0.74
**Insurance type**						
URRBMI	521	23.3	21.3	ref	ref	ref
UEBMI	1,943	15.9	16.2	0.71^***^	0.64	0.80
**Region**						
Eastern	1,107	17.6	17.4	ref	ref	ref
Central	824	17.9	18.1	1.05	0.95	1.16
Western	533	15.0	15.2	0.85^**^	0.76	0.95

*Abbreviations*: *PIM* potentially inappropriate medication, *OR* odds ratio, *95% CI* 95% confidence interval, *URRBMI* Urban Rural Resident Basic Medical Insurance, *UEBMI* Urban Employee Basic Medical Insurance

^a^adjusted by generalized linear model with logit link function and binomial distribution, using marginal standardization approach

^*^p < 0.05

^**^p < 0.01

^***^p < 0.001

### Cognition-impairing PIMs for older adults with PD

Among all older adults with PD, 6,201 (42.9%) had at least one prescription containing PIMs that could exacerbate cognitive impairment. Similarly, PD patients who were 65–74 years of age had a higher odds of receiving PIMs (44.8%, reference group), compared with patients who aged 75–84 (41.3%, OR, 0.86; 95% CI, 0.81–0.93) or > 84 (41.6%; OR, 0.88; 95% CI, 0.77–1.00) years. Patients who were male (OR, 0.66; 95% CI, 0.62–0.71), covered by UEBMI (OR, 0.61; 95% CI, 0.56–0.67), and residing in the western region (OR, 0.86; 95% CI, 0.79–0.93) were significantly associated with decreased odds of receiving cognition-impairing PIMs (Table 3). The rate of older PD persons being exposed to cognition-impairing PIMs, as evaluated by the 2023 Beers Criteria, was slightly lower than that observed when using the 2015 version (39.1% vs. 42.9%), which could be contributed to the removal of H2-receptor antagonists as cognitive-impairing PIMs, an update in the 2023 Beers Criteria.

**Table 3:** Factors associated with receipt of any cognition-impairing PIMs among older adults with Parkinson’s disease in China, 2015–2017 (*n* = 14,452)

Characteristic	PIM exposure	PIM rate (%)	Adjusted rate ^a^	OR	95% CI	
**Total**	6,201	42.9	42.9	-	-	-
**Age**						
65–74	3,000	45.4	44.8	ref	ref	ref
75–84	2,732	40.7	41.3	0.86^***^	0.81	0.93
≥ 85	469	41.0	41.6	0.88^*^	0.77	1.00
**Gender**						
Female	3,165	49.3	42.9	ref	ref	ref
Male	3,036	37.8	38.4	0.66^***^	0.62	0.71
**Insurance type**						
URRBMI	1,244	55.6	53.0	ref	ref	ref
UEBMI	4,957	40.6	42.9	0.61^***^	0.56	0.67
**Region**						
Eastern	2,751	43.7	43.5	ref	ref	ref
Central	2,047	44.5	44.5	1.05	0.97	1.13
Western	1,403	39.4	39.8	0.86^***^	0.79	0.93

*Abbreviations*: *PIM* potentially inappropriate medication, *OR* odds ratio, *95% CI* 95% confidence interval, *URRBMI* Urban Rural Resident Basic Medical Insurance, *UEBMI* Urban Employee Basic Medical Insurance

^a^adjusted by generalized linear model with logit link function and binomial distribution, using marginal standardization approach

^*^p < 0.05

^**^p < 0.01

^***^p < 0.001

### Distribution of PIMs by medications

Of all sampled patients, 4,826 (33.4%) patients were exposed to PIMs encompassing medications for the central nervous system, followed by anticholinergics (3,396 [23.5%]). Moreover, most medications for the nervous system and anticholinergics were also identified as motor- or cognition-impairing PIMs for PD patients. We list PIMs whose prevalence was over 0.5% in Table 4. Flupentixol/melitracen combination (794 [5.5%]) was the most prevalent PIM that may exacerbate motor symptoms in PD patients, followed by olanzapine (543 [3.8%]) and risperidone (539 [3.7%]), both of which are atypical antipsychotics commonly prescribed for patients with Parkinson's disease psychosis (PDP). Trihexyphenidyl (2,386 [16.5%]) was identified as the most prevalent PIMs associated with cognition- impairing drug-PD interactions, followed by estazolam (1,126 [7.8%]) and alprazolam (888 [6.1%], Table 4).

**Table 4:** Most common PIMs among older adults with Parkinson’s disease in China, 2015–2017

Organ System / Therapeutic Category / Generic	Patients, n (%)	Motor impaired PIM	Cognitive impaired PIM
***Central nervous system medications***			
**Antipsychotics**			
Olanzapine^a^	543 (3.8)	Yes	Yes
Risperidone	539 (3.7)	Yes	Yes
Perphenazine^a^	215 (1.5)	Yes	Yes
Chlorpromazine^a^	114 (0.8)	Yes	Yes
Haloperidol	109 (0.8)	Yes	Yes
Sulpiride	90 (0.6)	Yes	Yes
**Benzodiazepines**			
Eszolam	1126 (7.8)		Yes
Alprazolam	888 (6.1)		Yes
Clonazepam	420 (2.9)		Yes
Diazepam	414 (2.9)		Yes
Lorazepam	248 (1.7)		Yes
Oxazepam	94 (0.7)		Yes
**Non-benzodiazepines**			
Zolpidem	264 (1.8)		Yes
Eszopiclone	170 (1.2)		Yes
**Antidepressants**			
Flupentixol/Melitracen	794 (5.5)	Yes	Yes
Sertraline	286 (2.0)		
Escitalopram	225 (1.6)		
Paroxetine^a^	215 (1.5)		Yes
Fluoxetine	104 (0.7)		
Venlafaxine	92 (0.6)		
Citalopram	90 (0.6)		
Mirtazapine	86 (0.6)		
**Antiepileptics**			
Carbamazepine	87 (0.6)		
**Antiemetics**			
Metoclopramide	420 (2.9)	Yes	
Promethazine^a^	231 (1.6)	Yes	Yes
***Anticholinergics***			
Trihexyphenidyl	2386 (16.5)		Yes
***Cardiovascular medications***			
**Diuretics**			
Furosemide	1000 (6.9)		
***Endocrine system medications***			
**Sulfonylureas, long acting**			
Glimepiride	202 (1.4)		

^a^Drugs also with strong anticholinergic properties which not listed in *Anticholinergics class*

## Discussion

We conducted a cross-sectional analysis of PIM use among older PD patients in China between 2015 and 2017 based on a national representative insurance database. We observed that 57.8% of older adults with PD were exposed to at least one PIM, among which 17.1% and 42.9% were exposed to PIMs that could exacerbate their motor and cognitive impairments. Medications for the central nervous system and anticholinergics were most commonly used PIMs for older PD patients. Increasing age, female sex, being insured by URRBMI, and residing in the eastern or central regions were positively associated with the prevalence of PIM use.

The prevalence of PIM use among older PD patients was 57.8% in our study, which was much higher than that in the US (35.8%) [13], suggesting higher risks entailed in the pharmaceutical treatment for older PD patients in China. The prevalence of PIM use also differed between PD patients and the general population in China, with the latter being 29% as reported by a previous meta-analysis [28]. A total of 17.1% of sample patients had at least one PIM prescription that could aggravate their PD-related motor symptoms, which is double the estimate seen in a previous study in the US (8.7%) [13]. Our findings also revealed that 42.9% of older PD patients were exposed to at least one prescription containing PIMs that could exacerbate their cognitive impairment, also much higher than that in the US (29.0%) [13]. The potential cause of these discrepancies might be that pharmaceutical treatments for common cognitive symptoms of PD (e.g., antipsychotics for psychosis and benzodiazepines for insomnia) usually are PIMs that entail high risks of motor and/or cognitive impairment.

Most of the motor-impairing PIMs identified in our study are antipsychotic agents [29], which might also impair patients’ cognition. This is in line with the high prevalence of psychosis, with up to 60% of PD patients suffering from it [30]. Quetiapine and clozapine were the only two antipsychotics recommended for PDP by the AGS Beers criteria [12]. However, the most commonly used atypical antipsychotics in PD patients were olanzapine and risperidone in our study. As a previous study showed that olanzapine and risperidone were the top two consumed antipsychotics in China [31], this may indicate that clinicians did not differentiate between treatments of psychosis in the general population and PD patients. Besides, trihexyphenidyl, an anticholinergic anti-PD drug, was also a commonly used PIM, though it should be avoided in patients aged 60 and above for its possible cognitive impairment, as suggested by both the AGS Beers criteria and the Chinese guidelines for PD management [9, 32, 33]. Nevertheless, 16.5% of older adults with PD used trihexyphenidyl in our study, which was even higher than that among patients aged under 50 and with young-onset PD in China (14%) [34].

Our study also revealed that female PD patients were at greater risks of receiving PIMs than males, which was consistent with previous studies conducted in PD patients and in the general population [35, 36]. Epidemiological evidence indicated that affective disorders were more prevalent as comorbidities in female PD patients [37]. Therefore, antidepressants and benzodiazepines (BZDs) were more commonly used among females [38], which might partially explain the greater odds of receiving motor- and cognition-impairing PIMs of females. Besides, pharmaceutical treatment for older adults should be more cautious as aging is inextricably linked to the alterations in pharmacokinetics and pharmacodynamics, which may exacerbate drug-related problems [39, 40]. In this study, the overall prevalence of PIM use increased with age, however, the prevalence of motor- and cognition-impairing PIMs showed an inverse association with age. This may indicate that clinicians took more caution when prescribing medications likely to cause motor or cognitive impairments for older persons with PD.

Our results imply that medications’ potential harms or contra-indications for older adults with PD could to some extent be easily overlooked. Efforts shall be made to assess the appropriateness of medications prescribed for this already vulnerable population. For example, educational programs can keep clinicians updated about PIMs and competent in deciding the most appropriate mediations for older adults with PD. In other hand, prescribers are often well intentioned in their treatment but sometimes patients have symptoms that require treatment even if it comes with some degree of risks. For example, patients with behavioral issues that risk self-harm may require antipsychotics even if it will blunt the effects of their PD therapy. Pharmacists should be proactively involved in assessing medications prescribed as pharmaceutical therapy plays a cardinal role in PD treatment. Moreover, an expert guidance or consensus on medications to protect cognitive function of PD patients can also help clinicians better prescribe medications, which would in turn alleviate the burden of disease and health care expenditures [41–43]. For example, quetiapine and clozapine, which have priority over other antipsychotics for patients with PD, could be recommended as the first choice for controlling their psychosis symptoms.

To our best knowledge, this is the first study to analyze the prevalence and risk factors of PIM use among older adults with PD in China using a nationally representative health insurance database. The present study focused not only on the prevalence of general PIMs that pose potential risks for all older individuals but also on PD disease-specific PIMs. It’s worth noting that PIMs posing motor-impairment and cognition-impairment risks contributed greatly to the overall PIM use among older PD patients, which is in urgent need for clinical intervention. Several limitations of this study should be noted. First, due to the lack of information about the patients’ creatinine clearance, drug doses, and duration in our database, we excluded some PIMs listed in the 2015 AGS Beers criteria (Table S1). Therefore, the overall prevalence of PIM use may be underestimated. Second, owing to the limitations of the database, many factors that might have affected clinicians’ prescribing and drug choice, such as patients’ medical history, cognitive status, economic status, and long-term treatment patterns were not measured. Therefore, we were unable to identify an exhaustive list of predictors of PIM use or and clarify their mechanisms of action. Third, we did not include Traditional Chinese Medicines (TCMs) in our study because the Beers criteria did not contain recommendations for TCMs.

## Conclusion

More than half of older adults with PD in China received at least one PIM, especially for central nervous system medications and anticholinergics that pose particularly high risk*.* Females at advanced age were at a particularly high risk to the exposure of PIMs. More studies are needed to understand the rationale for, clinical impact of, and alternatives to PIMs commonly used in older adults with PD.

### Supplementary Information

**Additional file 1:** **Table S1.**Items of the Beers Criteria that excluded from study analyses. **Table S2. **Potentially inappropriate medications for PD patients due to motor impairment. **Table S3.**Potentially inappropriate medications for PD patients due to cognitive impairment. **Table S4. **Geographical division of China. **Table S5.**Overall PIM use of older patients with Parkinson’s disease in China, 2015-2017 using Beers criteria, 2023 version.

## Data Availability

The data that support the findings of this study are available from China Health Insurance Association but restrictions apply to the availability of these data, which were used under license for the current study, and so are not publicly available. Data are however available from the corresponding author (Xiaodong Guan) upon reasonable request and with permission of China Health Insurance Association.
