# Supplementary material for: Potentially inappropriate medications among older patients with Parkinson’s disease: a cross-sectional analysis of a national health insurance database in China
Source: BMC Geriatr. 2023 Dec 8;23:827. doi: 10.1186/s12877-023-04547-0 (PMC10709967; doi:10.1186/s12877-023-04547-0)
Supplement: Supplementary file 1 — Additional file 1: Table S1.Items of the Beers Criteria that excluded from study analyses. Table S2. Potentially inappropriate medications for PD patients due to motor impairment. Table S3.Potentially inappropriate medications for PD patients due to cognitive impairment. Table S4. Geographical division of China. Table S5.Overall PIM use of older patients with Parkinson’s disease in China, 2015-2017 using Beers criteria, 2023 version. [file 12877_2023_4547_MOESM1_ESM.docx]

Potentially inappropriate medications among older patients with Parkinson’s disease: a cross-sectional analysis of a national health insurance database in China

## Additional files

**Table S1.** Items of the Beers Criteria that excluded from study analyses

| **Medication(s)** | **Reason for exclusion** |
| --- | --- |
| Clozapine | These were among the safest choices for individuals with PDP |
| Quetiapine |  |
| H2-receptor antagonists | Weak evidence and to avoid overly restricting therapeutic options for older adults with dementia who have gastroesophageal reflux or similar issues |
| Nitrofurantoin | Creatinine clearance information not available |
| Dronedarone | Complete diagnoses information not available |
| Digoxin  Dabigatran  Rivaroxaban |  |
| Amiodarone |  |
| Chlordiazepoxide |  |
| Desmopressin |  |
| Vasodilators |  |
| Testosterone |  |
| Reserpine | Dose information not available |
| Doxepin |  |
| Metoclopramide | Duration information not available |
| Proton-pump inhibitors |  |
| Insulin, sliding scale | Unable to distinguish it from scheduled dosing |

* PDP, Parkinson's disease psychosis

**Table S2.** Potentially inappropriate medications for PD patients due to motor impairment

| **Classification** | **Medication** |
| --- | --- |
| Antipsychotics | Aripiprazole, Asenapine, Chlorpromazine, Droperidol, Fluphenazine, Haloperidol, Iloperidone, Loxapine, Lurasidone, Molindone, Olanzapine, Paliperidone, Perphenazine, Pimozide, Risperidone, Thioridazine, Thiothixene, Trifluoperazine, Ziprasidone |
| Antiemetics | Metoclopramide, Prochlorperazine, Promethazine |

**Table S3.** Potentially inappropriate medications for PD patients due to cognitive impairment

| **Classification** | **Medication** |
| --- | --- |
| Anticholinergics | Brompheniramine, Carbinoxamine, Chlorpheniramine, Clemastine, Cyproheptadine, Dexbrompheniramine, Dexchlorpheniramine, Dimenhydrinate, Diphenhydramine*, Doxylamine, Hydroxyzine, Meclizine, Triprolidine, Benztropine, Trihexyphenidyl, Cyclobenzaprine, Orphenadrine, Amitriptyline, Amoxapine, Clomipramine, Desipramine, Doxepin, Imipramine, Nortriptyline, Paroxetine, Protriptyline, Trimipramine, Chlorpromazine, Clozapine, Loxapine, Olanzapine, Perphenazine, Thioridazine, Trifluoperazine, Disopyramide, Darifenacin, Fesoterodine, Flavoxate, Oxybutynin, Solifenacin, Tolterodine, Trospium, Atropine#, Belladonna, alkaloids, Clidinium-chlordiazepoxide, Dicyclomine, Homatropine#, Hyoscyamine, Propantheline, Scopolamine#, Prochlorperazine, Promethazine |
| Benzodiazepines | Alprazolam, Chlordiazepoxide, Clobazam, Clonazepam, Clorazepate, Diazepam, Estazolam, Flurazepam, Lorazepam, Midazolam, Oxazepam, Temazepam, Triazolam, Quazepam |
| Nonbenzodiazepine, benzodiazepine receptor agonist hypnotics | Eszopiclone, Zaleplon, Zolpidem |
| Antipsychotics | Aripiprazole, Asenapine, Chlorpromazine, Droperidol, Fluphenazine, Haloperidol, Iloperidone, Loxapine, Lurasidone, Molindone, Olanzapine, Paliperidone, Perphenazine, Pimozide, Risperidone, Thioridazine, Thiothixene, Trifluoperazine, Ziprasidone, Flupentixol, Sulpiride |

*Oral route of administration

#Excluding ophthalmic

**Table S4.** Geographical division of China

| **Regions** | **Provinces** |
| --- | --- |
| East | Beijing, Tianjin, Hebei, Liaoning, Shanghai, Jiangsu, Zhejiang, Fujian, Shandong, Guangdong, Hainan |
| Central | Shanxi, Heilongjiang, Jilin, Anhui, Jiangxi, Henan, Hubei, Hunan |
| West | Chongqing, Sichuan, Guizhou, Yunnan, Tibet, Shaanxi, Gansu, Ningxia, Qinghai, Xinjiang, Guangxi, Inner Mongoria |

**Table S5.** Overall PIM use of older patients with Parkinson’s disease in China, 2015-2017 using Beers criteria, 2023 version

| **Characteristic** | **Sample size (n, %)** | **PIM exposure** | **PIM rate (%)** | **Adjusted rate ^a^** | **OR** | **95% CI** | |
| --- | --- | --- | --- | --- | --- | --- | --- |
|  | 14,452 (100.0) | 9,160 | 63.4 | 63.4 |  | - | - |
| **Age** |  |  |  |  |  |  |  |
| 65-74 | 6,603 (45.7) | 4,023 | 60.9 | 60.5 | ref. | ref. | ref. |
| 75-84 | 6,705 (46.4) | 4,398 | 65.6 | 66.0 | 1.27*** | 1.18 | 1.36 |
| ≥85 | 1,144 (7.9) | 739 | 64.6 | 64.7 | 1.20*** | 1.05 | 1.37 |
| **Gender** |  |  |  |  |  |  |  |
| Female | 6,415 (44.4) | 4,260 | 66.4 | 65.9 | ref. | ref. | ref. |
| Male | 8,037 (55.6) | 4,899 | 61.0 | 61.4 | 0.82*** | 0.76 | 0.88 |
| **Insurance type** |  |  |  |  |  |  |  |
| URRBMI | 2,237 (15.5) | 1621 | 72.4 | 71.8 | ref. | ref. | ref. |
| UEBMI | 12,215 (84.5) | 7,539 | 61.7 | 61.9 | 0.63*** | 0.57 | 0.70 |
| **Region** |  |  |  |  |  |  |  |
| East | 6,292 (43.5) | 4108 | 65.3 | 65.2 | ref. | ref. | ref. |
| Central | 4,596 (31.8) | 3040 | 66.1 | 65.9 | 1.03 | 0.95 | 1.12 |
| West | 3,564 (24.7) | 2012 | 56.5 | 57.0 | 0.70*** | 0.65 | 0.77 |

Abbreviations: PIM, potentially inappropriate medication; OR, Adjusted odds ratio; 95% CI, 95% confidence interval; URRBMI, Urban and Rural Resident Basic Medical Insurance; UEMI, Urban Employee Basic Medical Insurance

a. adjusted by generalized linear model with logit link function and binomial distribution, using marginal standardization approach

* *p* < 0.05, ** *p* < 0.01, *** *p* < 0.001
